# Effects of social capital and technology cognition on farmers' adoption of soil and water conservation tillage technology in the Loess Plateau of China

**DOI:** 10.1016/j.heliyon.2024.e27137

**Published:** 2024-02-28

**Authors:** Liu Li, Shangguan Dingyi, Sun Fengluan, Tai Xiujun, Hafeez Noor

**Affiliations:** a School of Economics and Management, Shanxi Normal University, Taiyuan 030000, China; b College of History and Tourism Culture, Shanxi Normal University, Taiyuan 030000, China; c College of Agriculture, Shanxi Agriculture University, Taigu 030801, Shanxi, China

**Keywords:** Social capital, Technology cognition, Technology adoption, Regional differences, Multi-group structural equation model

## Abstract

From the aspect of regional differences, this paper investigated the impact of social capital and technology cognition on the adoption of soil and water conservation tillage technology in the Loess Plateau in China. We find social networks and social trust had significant impact on the adoption of contour tillage technology by farmers in Shanxi and Shaanxi. Social participation had a significant impact in Shaanxi, whereas social prestige had a significant impact in Gansu, and social norms had a significant impact on the adoption of contour tillage technology in the three provinces. Technology cognition played an intermediary role in the effects of social networks, social trust, and social norms on technology adoption in Shaanxi and Shanxi, and on the impact of social norms on technology adoption in Gansu. Considering social networks, the frequency of communication between farmers and villagers had the greatest impact on technology adoption in Shanxi and Shaanxi, while farmers' trust in villagers had the greatest impact on technology adoption in these two provinces. The participation in collective activities in the village had the greatest impact on technology adoption in Shaanxi. Furthermore as for social prestige there was little difference in the degree of impact of observation variables on technology adoption by farmers in Gansu. Finally, regarding social norms, the attitudes and behaviors adopted by relative's friends, and villagers in the village had the greatest impact.

## 1 Introduction

Since the 18th National Congress of the Communist Party of China (CPC), construction of ecological civilization has attracted considerable attention, as the central and local governments have introduced a series of policies. Ecological civilization construction is mainly aimed at solving problems threatening the harmonious coexistence of humans and nature (e.g., resource shortage, ecological degradation, and environmental pollution). China has constructed ecological civilization by optimizing the pattern of territorial development, promoting resource conservation, intensifying the protection of natural ecosystems and the environment, and strengthening institutional building. Ecological degradation (soil erosion, desertification, rocky desertification, and marine ecological degradation) is a major problem in the process of ecological civilization construction, among which soil erosion is particularly prominent. Currently, the area of soil and water loss in China is as high as 2,949,100 km^2^. In the Loess Plateau, each year about 900–1100 km^2^ of soil and water is lost due to human activity, with up to 1.6 billion tons of sediment entering the Yellow River (Ministry of Water Recourses of the People's Republic of China, 2018). Soil and water loss leads to deterioration of ecological environment; furthermore, it lowers land productivity and degrades social economy. In the case of climate change, global warming causes changes in rain capacity and its characteristic [1,2]. These changes affect significantly the soil erosion and runoff in the Loess Plateau.

Over the years, a series of measures have been adopted to control soil and water loss, including engineering, biological, and tillage measures [3,4]. According to the strategic plan for rural revitalization (2018–2022) issued by the central committee of CPC and the state council, soil and water conservation should be promoted by respecting the farmers’ wishes. In the implementation of engineering measures (ditch, slope, drainage) and planting measures (afforestation and grass planting, turning farmland into forest), government is the key investor and main actor, and farmers are mere passive participants. As for soil and water conservation tillage technology, farmers are the main actors, and they choose and adopt technology according to their needs, while governments are mainly responsible for technology promotion services. Compared to traditional technology, soil and water conservation tillage technology significantly reduces runoff erosion, improves soil quality, and increases agricultural yield. Therefore, in the process of ecological civilization construction, application and popularization of soil and water conservation tillage technology have become an inevitable choice in controlling soil erosion.

As a supporting measure of slope modification engineering, contour tillage technology plays an important role in soil and water loss control of slope farmland on the Loess Plateau. Due to the small land area of terrace and slope, large machinery cannot operate, the lack of small machinery, resulting in mechanized operation is difficult, farmers generally believe that the convenience of technology is poor, reluctant to invest labor and time. In the investigation, it was found that many farmers in the Loess Plateau area abandoned their terraced fields, and the adoption rate of contorur tillage technology was very low.

Studies on adoption behavior regarding the soil and water conservation technology have been conducted by domestic and foreign scholars, considering how personal characteristics (e.g., gender, age, education, psychological traits, etc.) [[5], [6], [7]]. Family characteristics (e.g., income level, income structure, labor force and resource endowment, etc.) [[8], [9], [10], [11]], and public policy, including government subsidies (technology promotion, training, etc) [[12], [13], [14]]. Affect farmers' technology adoption behavior. Recently, some scholars have paid attention to the impact of land transfer on farmers’ technology adoption [[15], [16], [17]].

Social capital is broadly defined as a valuable asset based on inter-personal social relationship [18]. In China, farmers obtain information and resources from family members, relatives, and friends; local communities and so forth, because their lives and agricultural production are relatively concentrated. Thus, we define the aforementioned factors that affect farmers' behaviors as social capital. However, as rational persons farmers do not blindly follow the group. Thus, when adopting soil and water conservation tillage technology, they will also consider features, functions inputs, and effects. In this paper, technology cognition is defined as the understanding of technology after contacting and learning relevant information, including technology profitability, effectiveness, and convenience, which affects their willingness to adopt technology, consequently, their ultimate decisions. The existing studies mainly focus on perceived ease of use and perceived usefulness [[19], [20], [21]]. Nevertheless, we should note that farmers’ technology cognition includes not only cognition of function and effect, but also risks.

Based on previous studies, we divide social capital into social networks, social trust, social participation, social prestige, and social norms. In addition, we consider farmers' technology cognition including the perspective of technology convenience, technology effect, and technology risk. Subsequently, we put social capital and technology cognition in a theoretical framework to study their impact on the farmers' adoption of soil and water conservation tillage technology. Moreover, there are significant regional differences in technology adoption due to the vastness of the Loess Plateau. Therefore, from the perspective of regional differences, taking contour tillage as an example, we use a multi-group structural equation model to explore the impact of social capital and technology cognition on the adoption of soil and water conservation tillage technology by farmers, providing a scientific basis for the further technology promotion. Considering the background of global climate change, the harm of soil erosion in the Loess Plateau has become an unavoidable problem in China. Especially for farmland soil erosion and food security, it is necessary to pay attention to farmers' adoption of soil and water conservation tillage technology. This can help governments and related sectors to fully grasp the farmers’ willingness to adopt the soil and water conservation tillage technology, and identify the key factors, thus formulating and implementing relevant policies and measures to promote soil and water conservation tillage technology adoption, and finally improving ecological environment.

## 2 Theoretical basis and research hypothesis

### 2.1 The impact of social capital on farmers’ adoption of soil and water conservation tillage technology

Social capital is relative to material capital and human capital, as it broadly refers to interpersonal relationships, including social networks, social norms, social authority, consensus on action, social morality, social trust, and other aspects [22]. It is an intangible resource, being the connection between individuals and others, or organizations that impact their behaviors [23,24]. The more social capital individuals have, the more information and opportunities they can acquire, thus reaping certain benefits [25,26].

In this paper, we measure social capital based on five perspectives, namely, social networks, social trust, social participation, social prestige, and social norms. Individuals can obtain useful information, experience, technology, capital, and other resources from their social networks [[27], [28], [29]]. Which will impact their behaviors and decisions. The farmers’ social trust is reflected in their willingness to believe or follow the advice of others to make decisions [30]. Social participation can enable farmers to integrate into their respective groups, obtain more social resources and information, and so forth, which is conducive to a more rational decision. The higher the social prestige of farmers, the higher their status in the village and the greater their decision-making impact on others; subsequently, the more resources they can grasp, and the stronger its ability to shape and help in public affairs [31]. Individuals will perceive the pressure or expectation of a social group and internalize social norms into the moral norm and code of conduct for their own actions through unconscious or conscious learning, and then act in accordance with social norm. As for soil and water conservation tillage technology, when a farmer sees that most farmers are adopting, he feels pressure or expectations from surrounding people, thus adopt such technology.

Social capital can enhance the sense of cooperation among farmers and support the supply of public goods and their collective actions [[32], [33], [34], [35]]. Although adoption of soil and water conservation tillage technology is a process of the farmer's independent decision, the technology itself is similar to public goods, and it can bring social and ecological benefit. As for the adoption of soil and water conservation tillage technology, social networks, social trust, social participation and social prestige bring more information and resources for farmers, enhancing understanding of technology. Social norms prevent them from doing anything harmful to soil and water conservation.

Based on the aforementioned analysis, the following hypotheses are proposed.

H1 Social capital can promote farmers' adoption of soil and water conservation tillage technology. The more social capital individuals have, the more information and opportunities they can acquire, thus making rational decisions.

H1a The larger the social networks scale, the more resources and technology information can be obtained from social networks, which is helpful for farmers to make correct technology adoption decisions.

H1b Social trust can reduce the time and risk for farmers to search for information. The more farmers trust the surrounding population, the more dependent they are on the technology information obtained from it, which is conducive to technology adoption.

H1c Farmers can obtain technology information by participating in collective affairs in the village or in activities with their neighbors. The more frequent communication among farmers, the more technology information they get; thus, social participation promotes the adoption of technology.

H1d The higher the social prestige, the higher the status in the village, and the greater the impact of their technology adoption decisions on others.

H1e Farmers' technology adoption behavior will be influenced by the attitude of people around them, which will be transformed into their own code of conduct. Therefore, social norms affect farmers' technology adoption.

### 2.2 The mediating effect of technology cognition between social capital and conservation tillage technology adoption

Technology cognition is the primary link of technology adoption [36]. Studied the innovative technology adoption and defined technology cognition as the nominal familiarity and contact with innovative technology [37]. Defined technology cognition as the process of someone's effort to collect and understand technology information. Social capital can be used to disseminate information. Information dissemination brings technology knowledge to farmers, improves their technology cognition and thus increases the rate of technology adoption [38]. With the increase of the number of farmers adopting the technology, the accumulation of technical information will further improve the cognition of farmers. The learning effect is manifested in that farmers who have not adopted the technology will consult those who have adopted the technology, and the diffusion efficiency of the technology will be improved, making the new technology familiar to farmers [18]. In addition, the communication between farmers can enable them to better grasp the operation methods of technology, which is conducive to the play of technical effects, and farmers are more willing to adopt technology [39]. Social capital can promote the cognitive level of farmers, which is conducive to the generation of behaviors [40]. The more abundant social capital, the higher the cognition level of farmers' cultivated land value, which ultimately prompts farmers to protect cultivated land, and cognition plays an intermediary role between social capital and farmers' behavior.

Based on the aforementioned analysis, the following hypotheses are proposed.

H2 Technology cognition plays an intermediary role in the impact of social capital on the adoption of soil and water conservation tillage technology.

H2a Technology cognition plays an intermediary role in the impact of social networks on the adoption of soil and water conservation tillage technology.

H2b Technology cognition plays an intermediary role in the impact of social trust on the adoption of soil and water conservation tillage technology.

H2c Technology cognition plays an intermediary role in the impact of social participation on the adoption of soil and water conservation tillage technology.

H2d Technology cognition plays an intermediary role in the impact of social prestige on the adoption of soil and water conservation tillage technology.

H2e Technology cognition plays an intermediary role in the impact of social norms on the adoption of soil and water conservation tillage technology.

The Loess Plateau region includes 287 counties in seven provinces, with significant differences in farming systems among different regions [41]. Due to the differences in natural, geographical, and economic conditions in various regions, farmers’ social capital and technology cognition are different, thus affecting their technology adoption.

## 3 Material and methods

### 3.1 Study area

**Fig. 1:**
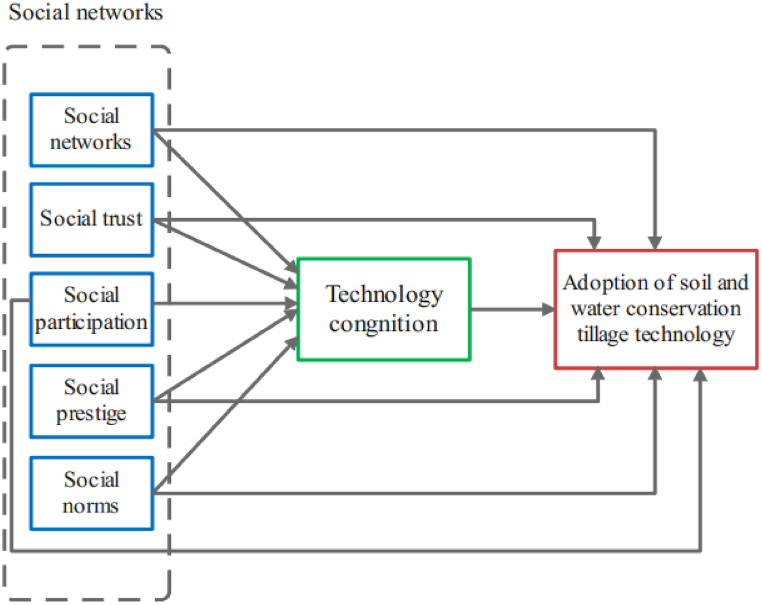
Theoretical framework.

The Loess Plateau covers a vast territory, mainly including 287 counties in Shanxi, Shaanxi, Gansu, Qinghai, Ningxia, Henan, and some other provinces. It covers an area of 400,000 km^2^, accounting for 70% of the loess distribution in the world, and it is the largest loess accumulation area in the world. The climate is relatively dry, precipitation is concentrated, vegetation is sparse, and soil erosion is serious. The average annual rainfall of the Loess Plateau is 466 mm, and the flat cultivated land is generally less than 1/10. Most of the cultivated land is distributed on slopes of approximately 10°–35°, and the plots are narrow and scattered. As a result of temperature, moisture, and geomorphology difference, it formed different tillage systems (see Fig. 1).

**Fig. 2:**
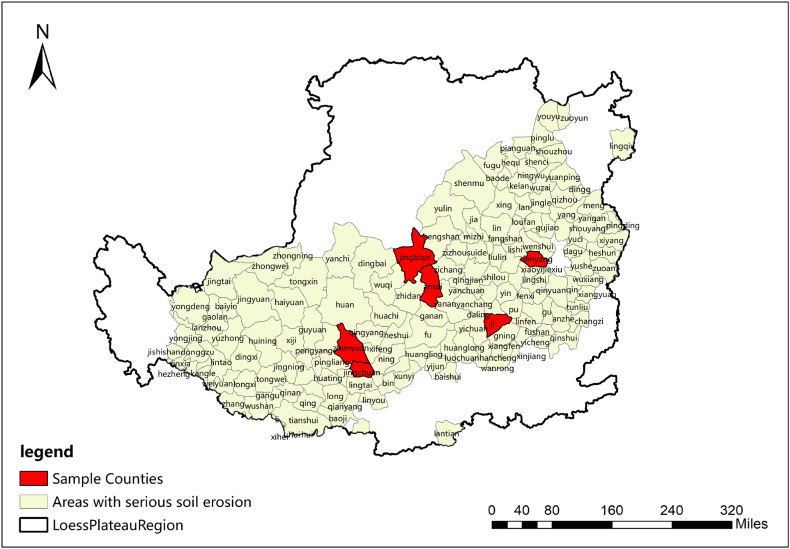
Study area and sample counties.

We investigate the most serious soil erosion in the Loess Plateau. In the study area, we selected Fenyang city and Ji county of Shanxi Province in the Fen Wei plain sub-humid area, Ansai County and Jingbian county of Shaanxi Province in the loess hilly region, and Zhenyuan County and Jingchuan county of Gansu Province in the residual tableland Loess region (Fig. 2.). The three regions (provinces) are located in the east, in the center, and the west of the Loess Plateau. Terrain, rainfall, the level of economic development, and planting structure are similar within the region, in addition to obvious differences between the regions.

### 3.2 Data sources

We surveyed farmers from January to March 2019, using the stratified random sampling method to determine the cities and counties with the most serious soil and water loss, according to the literature. Two counties were selected from each province, two to three towns were selected from each county, three to five villages were randomly selected from each town, and 15–25 farmers were randomly selected from each village. The investigators of the research group used field investigation to randomly enter the households of farmers to investigate. Mainly investigating decision makers among family members (head of household), due to the special situation in China, the head of household is generally male, so in the 1237 respondents, there are 1171 men and only 66 women. In the survey, about 90% of farmers were willing to be interviewed, and a few farmers were reluctant to cooperate because their families did not farm. Farmers who do not farm at home do not use technology and therefore are not covered by the survey. Sample distribution and technology adoption are shown in Table 1. We surveyed 1316 farmers, obtaining 1237 responses, with an effective response rate of 94%. Descriptive statistics of the samples are shown in Table 2.

**Table 1:** Sample distribution and technology adoption.

Province	County	Township (town)	Number of Samples	Percentage (%)	Number of Technology Adoptions
Shanxi	Ji county	Tunli town, Chengguan town, Baishansi township	244	19.73	191
	Fenyang city	Yanwu town, Xiaojiazhuang town	196	15.84	
Shaanxi	Ansai county	Zhenwudong town, Yanhewan town, Huaziping town	215	17.38	179
	Jingbian county	Dongkeng town, Yangshupan town, Longzhou town	203	16.42	
Gansu	Zhenyuan county	Chengguan town, Tunzi town, Shangxiao township	197	15.92	69
	Jingchuan county	Chengguan town, Yvdu town,	182	14.71	
Total	–	–	1237	100.00	439

**Table 2:** Descriptive statistics of samples.

Variable	Meaning	Assignment	Min	Max	Mean	Standard Deviation
Age	Age of the head of household	age	26	83	55.35	10.669
Education	Education of the head of household	1 = untaught 2 = primary school 3 = junior school 4 = high school 5 = college or above	1	5	2.47	0.887
Village cadre	The head of household is a village cadre	0 = no; 1 = yes	0	1	0.07	0.262
Labor	Household labor force	quantity	0	8	2.27	1.077
Cooperative	Joined the agricultural machinery cooperative	0 = no; 1 = yes	0	1	0.08	0.269
Distance from village to county	Distance from village to county	li	1.0	125.0	19.113	14.6630
Technology promotion	How many soil and water conservation tillage technology promotion services have you received?	quantity	1	5	3.9458	0.9293
Convenience of farm machinery	The easiness to obtain machines	1 = very difficult 2 = not so easy 3 = moderate 4 = easier 5 = easiest	1	5	2.6451	1.3944

### 3.3 Variables setting

#### 3.3.1 Core independent variables

Social capital is the independent variable, it is including social networks, social trust, social participation, social prestige, and social norms. They were measured using a five-point Likert scale (Table 3) (see Table 4).

**Table 3:** Variables and assignments.

Latent Variables	Observation Variables	Assignment
Social networks (SN)	SN1 Contact with relatives and friends	1 = never; 2 = rarely; 3 = moderately; 4 = frequently; 5 = very frequently
	SN2 Contact with villagers in this village	
	SN3 Contact with village cadres	
Social trust(ST)	ST1 Degree of trust in relatives and friends	1 = very distrustful; 2 = distrustful; 3 = neutral; 4 = trustful; 5 = very trustful
	ST2 Degree of trust in the villagers	
	ST3 Degree of trust in the village cadres	
Social participation (SP)	SP1 Joint entertainment with neighbors (playing cards or dancing, etc.)	1 = strongly disagree; 2 = disagree; 3 = neutral; 4 = agree; 5 = strongly agree
	SP2 Participate in group activities in the village	
	SP3 Vote in the elections of village cadres	
Social prestige (SR)	SR1 Are invited to attend when someone else has affairs	
	SR2 Relatives, friends, and neighbors come to help when the family is in trouble.	
	SR3 Ask you to help mediate conflicts.	
Social norms (SS)	SS1 Relatives and friends believe that the soil and water conservation tillage technology should be adopted.	
	SS2 Villagers believe that the soil and water conservation tillage technology should be adopted.	
	SS3 Village cadres believe that the soil and water conservation tillage technology should be adopted.	
Technology cognition (TC)	TC1 Technology is easy to use.	
	TC2 Technology has to be supported by machines and tools.	
	TC3 Technology can increase the output.	
	TC4 Technology can control soil erosion.	
	TC5 You can take the risk of adopting the technology.	
	TC6 Technology is suitable for local.	
Technology adoption	TA1 You adopt the contour tillage technology.	0 = no; 1 = yes
	TA2 You will continue to adopt contour tillage in the future.

**Table 4:** Reliability and validity test results.

Latent Variables	Observation Variables	Factor Loading	Cronbach's α
Social networks (SN)	SN1	0.836	0.861
	SN2	0.735	
	SN3	0.776	
Social trust (ST)	ST1	0.801	0.833
	ST2	0.834	
	ST3	0.628	
Social participation (SP)	SP1	0.813	0.817
	SP2	0.865	
	SP3	0.732	
Social prestige (SR)	SR1	0.739	0.905
	SR2	0.677	
	SR3	0.761	
Social norms (SS)	SS1	0.907	0.846
	SS2	0.877	
	SS3	0.827	
Technology cognition (TC)	TC1	0.815	0.938
	TC2	0.871	
	TC3	0.774	
	TC4	0.807	
	TC5	0.737	
	TC6	0.714	
Technology adoption (TA)	TA1	0.819	0.725
	TA2	0.819	

Social networks were measured by the frequency of contact with relatives, friends, villagers, and village cadres. Social trust was measured by the degree of trust in relatives, friends, villagers, and village cadres. Social participation was measured considering joint entertainment with neighbors, participation in collective activities in the village, and participation in the election of village cadres. Social prestige was measured by observation variables such as being involved in others’ affairs; relatives, friends, and neighbors coming to help in case of trouble; and helping to mediate conflicts. Social norms were measured by the attitudes of relatives, friends, villagers, and village cadres toward the adoption of soil and water conservation tillage technology.

#### 3.3.2 Intermediary variable

Technology cognition includes six items, among which “technology is easy to use” and “technology has to be supported by machinery and tools” represent the farmers' cognition of technology convenience; “technology can improve yield” and “technology can control soil erosion” are the farmers' cognition of technology effect; and “you can take the risk of adopting the technology” and “technology is suitable for local” are the farmers’ cognition of technology risks.

#### 3.3.3 Dependent variables

Technology adoption is a dependent variable. To evaluate the process of technology adoption, combined with the actual situation of the Loess Plateau, we selected contour tillage technology. Technology adoption is a binary variable, where 0 stands for “no adoption,” and 1 means “for adoption.”

### 3.4 Model setting

Social capital includes five latent variables social capital and technology cognition directly affect farmers’ adoption of soil and water conservation tillage technology. Simultaneously, technology cognition plays an intermediary role between social capital and technology adoption. In addition, this study considers different behaviors of farmers in different regions of the Loess Plateau. According to the research needs, we adopted the multi-group structural equation model.

First, we analyzed the validity and reliability of the questionnaire, in which Cronbach's Alpha value was used for reliability test, and KMO and Bartlett were used for validity test. Then, the goodness of fit of structural equation model was tested in the three provinces. Finally, path analysis was carried out including direct and indirect effects, and the impact path of observation variables on latent variables.

## 4 Results

### 4.1 Reliability and validity test

First, we carried out reliability test of farmers' questionnaire using SPSS 24.0, with an overall sample of Cronbach's Alpha value being 0.837, indicating high reliability of the questionnaire. For the test of questionnaire validity, the KMO and Bartlett tests showed that the KMO value was 0.823, being >0.5, and Bartlett's spherical test value was 16280.950, with a significance level of 0.000; thus we were able to extract common factor analysis. After factor analysis, factor loading coefficients of observation variables were all >0.5, with a cumulative variance contribution rate >70%, which could represent well corresponding latent variables.

### 4.2 Goodness of fit of model test

The goodness of fit of models in Shanxi, Shaanxi, and Gansu were tested, using AMOS 24.0. Absolute goodness, relative goodness, and simplified goodness of fit indexes were selected to evaluate the fitting effect of the models.

Table 5 shows that the fitting indexes of contour tillage technology adoption models are not above the threshold value, indicating that the models constructed are of good robustness. Therefore, they can be used to analyze technology adoption.

**Table 5:** Goodness of fit of multiple groups.

Statistical Test Index Type	Goodness of Fit Statistics	Shanxi	Shaanxi	Gansu	Norm
Absolute goodness of fit index	CMIN/DF	3.664	3.818	3.925	<5
	GFI	0.876	0.826	0.906	>0.8
	AGFI	0.856	0.835	0.872	>0.8
	RMR	0.032	0.012	0.015	<0.08
Relative goodness of fit index	CFI	0.854	0.870	0.891	>0.8
	NFI	0.832	0.850	0.871	>0.8
	TLI	0.802	0.843	0.860	>0.8
Simplified goodness of fit index	PGFI	0.623	0.702	0.548	>0.5
	PNFI	0.605	0.719	0.533	>0.5

### 4.3 Research hypotheses tests

#### 4.3.1 Direct effects test

AMOS 24.0 was used for the path analysis of the multi-group structural equation model, with the results being shown in Table 6.

**Table 6:** Multi-group hypothesis test.

Direct Effects Hypothesis	Shanxi	Shaanxi	Gansu
H1a:TA←SN	0.234**	0.148**	0.571
H1b:TA←ST	0.156*	0.359*	0.698
H1c:TA←SP	0.016	0.044*	0.148
H1d:TA←SR	0.023	0.248	0.097**
H1e:TA←SS	0.144***	0.103*	0.246***
H2: TA←TC	0.619***	0.708***	0.576***

Note: *, **, *** denote p < 0.05, p < 0.01, and ***p < 0.001.

**Table 7:** Intermediary effect of multi-group technology cognition.

Path	Shanxi			Shaanxi			Gansu		
	Direct effect	Intermediate effect	Total effect	Direct effect	Intermediate effect	Total effect	Direct effect	Intermediate effect	Total effect
TA←SN	0.234	0.032	0.266	0.148	0.134	0.282	–	–	–
TA←ST	0.156	0.263	0.419	0.359	0.225	0.548	–	–	–
TA←SP	–	–	–	0.044	–	0.044	–	–	–
TA←SR	–	–	–	–	–	–	0.097	–	0.097
TA←SS	0.144	0.279	0.423	0.103	0.328	0.431	0.246	0.215	0.461
TA←TC	0.619	–	0.619	0.708	–	0.708	0.576	–	0.576

The components of social capital had a direct positive impact on the adoption of soil and water conservation tillage technology by farmers (see Table 7). Social networks had a significant impact on the technology adoption in the Shanxi and Shaanxi, but not in Gansu. Social trust had a significant impact on the technology adoption in Shanxi and Shaanxi, but not in Gansu. Social participation had a significant impact on the technology adoption in Shaanxi province, but not in Shanxi and Gansu. Social prestige had a significant impact on the technology adoption in Gansu, but not in Shanxi and Shaanxi. Social norms had a significant impact on the technology adoption in all three provinces.

Technology cognition had a significant positive effect on farmers’ adoption of contour tillage technology in the three provinces, but the impact coefficient had a significant regional difference. The impact of technology cognition on the adoption of contour tillage technology was the greatest in Shaanxi Province, followed by Shanxi and Gansu.

Therefore, hypothesis H1 is partially supported, while hypotheseH2 is fully supported.

#### 4.3.2 Intermediate effects test

Technology cognition plays an intermediary role in the effect of farmers’ social capital on the adoption of soil and water conservation tillage technology. To verify intermediate effect, it needs to meet four conditions. First, independent variables can have a significant impact on dependent variable. Second, independent variables have a significant impact on intermediary variables. Third, intermediary variables have a significant effect on dependent variable. Finally, the impact of independent variables on the dependent variable is minor or not significant when considering intermediary variables. If the above four conditions cannot be simultaneously satisfied, then the intermediary effect does not exist. If intermediary effect does exists, the effects of impact coefficients of independent variables on intermediary variables and impact coefficients of intermediary variables on dependent variable can be used in calculation. Finally, total effect of the independent variables on dependent variable is the sum of the direct and intermediate effects. If direct effect is not significant, intermediate effect does not exist, and the total effect need not calculated. If direct effect is significant, and intermediate effect does not exist, then the total effect is equal to direct effect. According to the aforementioned conditions, this study combines Table 6, Table 8 to obtain the results of intermediary effects.

**Table 8:** Path coefficient estimation results.

Path	Shanxi		Shaanxi		Gansu	
	Unnormalized path coefficients	Normalized path coefficient	Unnormalized path coefficients	Normalized path coefficient	Unnormalized path coefficients	Normalized path coefficient
TC←SN	0.094	0.052*	0.220	0.189*	0.256	0.394**
TC←ST	0.334	0.425***	0.286	0.318**	0.170	0.205***
TC←SP	0.120	0.030	0.365	0.251	0.125	0.237*
TC←SR	0.072	0.136*	0.228	0.279	0.080	0.100
TC←SS	0.359	0.451***	0.397	0.463***	0.559	0.373***
SN1←SN	4.071	0.693**	1.240	0.557***	5.094	0.505***
SN2←SN	5.179	0.912**	1.594	0.827***	2.500	0.407***
SN3←SN	1.000	0.148	1.000	0.422	1.000	0.194
ST1←ST	1.853	0.884***	1.054	0.739***	1.100	0.895***
ST2←ST	2.171	0.943***	1.247	0.859***	1.111	0.859***
ST3←ST	1.000	0.333	1.000	0.579	1.000	0.574
SP1←SP	1.000	0.816	1.000	0.248	0.130	0.144*
SP2←SP	8.601	0.753	1.558	0.538**	0.768	0.540***
SP3←SP	9.496	0.054	1.084	0.324**	1.000	0.730
SR1←SR	1.296	0.563***	1.000	0.732	1.000	0.858
SR2←SR	1.000	0.722	1.276	0.784***	1.186	0.789***
SR3←SR	0.490	0.405***	0.880	0.650***	0.768	0.723***
SS1←SS	1.826	0.934***	1.036	0.851***	5.122	0.639***
SS2←SS	1.693	0.890***	1.085	0.878***	6.186	0.246**
SS3←SS	1.000	0.576	1.000	0.894	1.000	0.058
TC1←TC	1.000	0.384	1.000	0.637	1.000	0.096
TC2←TC	3.871	0.758***	2.048	0.754***	0.171	0.071*
TC3←TC	0.287	0.124**	1.917	0.757***	2.195	0.731***
TC4←TC	1.871	0.629***	0.997	0.609***	1.363	0.870***
TC5←TC	0.584	0.263***	2.069	0.769***	0.113	0.840***
TC6←TC	3.723	0.824***	1.921	0.663***	0.936	0.886***
TA1←TA	1.000	0.868	1.000	0.871	1.000	0.766
TA2←TA	2.950	0.653***	2.906	0.881***	2.542	0.693

Note: *, **, *** denote p < 0.05, p < 0.01, and ***p < 0.001.

In adoption of contour tillage technology by farmers in Shanxi and Shaanxi, technology cognition plays an intermediary role in the impact of social networks, social trust, and social norms on technology adoption. In adoption of contour tillage technology by farmers in Gansu province, technology cognition plays an intermediary role in the impact of social norms on technology adoption. Thus, hypothesis H3 is partially supported.

### 4.4 Path analysis

We used AMOS 24.0 to calculate the multi-group structural equation path, with the results presented in Table 8.

(1) Social networks and technology cognition have a significant positive impact on technology adoption in Shanxi, in addition, social networks have a significant positive impact on the adoption of contour tillage technology in Shaanxi. Technology cognition plays a positive intermediary role similar to the impact of farmers' social networks in the two provinces, with the intermediate effects being 0.032 and 0.134, respectively, with technology cognition of all observation variables, including technology convenience cognition, technology effect cognition, and technology risk cognition passing the significance test. The frequency of contact between farmers and villagers in the social networks has the greatest impact on technology adoption in Shanxi and Shaanxi, indicating that the farmers' technology adoption in the two provinces is most affected by the villagers, with the imitation effect being obvious. Social networks have no significant impact on adoption of contour tillage technology in Gansu. A possible reason is that Shanxi and Shaanxi provinces are located in the loess hilly region, where farmers' cultivated land includes a large area of slopes and terraces. For many years, contour tillage technology has played a key role in controlling soil erosion and improving agricultural production, with a relatively greater number of farmers adopting it. The larger the scale of social networks and the higher their intensity, the more resources and information can be accessed, and the more extensive technology guidance and demonstration can be received, thus enhancing farmers' technology cognition and promoting technology adoption. Conversely, the investigation area in the Gansu province includes Zhenyuan County and Jingchuan County, where the land cultivated by farmers is generally flat, and the contour tillage technology is adopted by fewer farmers, making the impact of social networks and technology cognition on technology adoption insignificant.
(2) Social trust and technology cognition had a significant positive impact on the adoption of contour tillage technology in Shanxi, with the standardized path coefficients of each observation variable being 0.884, 0.943, and 0.333, respectively, all passing the significance test. In addition, social trust had a significant positive impact in Shaanxi, with the standardized path coefficients of each observation variable being 0.739, 0.859, and 0.579, respectively, all of them passing the significance test. Technology cognition played a positive intermediary role, similar to the impact of farmers' social trust in the two provinces, with intermediate effects of 0.263 and 0.225, respectively, and with all observation variables including cognition of technology convenience, technology effect, and technology risk passing the significance test. Among observation variables of social trust, the degree of farmers' trust in the villagers had the greatest impact on adoption of contour tillage technology in Shanxi and Shaanxi. Moreover, social trust did not have a significant impact in Gansu. One possible reason is that in Shanxi and Shaanxi, where contour tillage technology is more prevalent, the stronger the farmers' social trust was, the higher the reliability of obtaining technology information from surroundings, which is conducive to stimulating farmers' technology adoption. Social trust can reduce time and risk of information search, as farmers can obtain reliable technology information from surroundings.
(3) Social participation has a significant positive impact on the adoption of contour tillage technology by farmers in Shaanxi, with standardized path coefficients of observation variables being 0.248, 0.538, and 0.324, all of them passing the significance test. Among the observation variables of social participation, participation in collective activities in villages has the greatest impact on the farmers' adoption of contour tillage technology. However, social participation did not have significant impact in Shanxi and Gansu. One possible reason is that Ansai and Jingbian counties in Shaanxi Province are the areas most affected by soil erosion in China, while contour tillage technology has been used since the 1950s with a remarkable effect on soil and water conservation and yield. Additionally, farmers have been participating in village collective activities such as returning farmland to forest program, building silt dams, and water conservancy. Farmers can obtain more technology information from village groups through social participation, thus improving the level of technology cognition. Moreover, government subsidies for contour tillage technology can promote farmers' technology adoption.
(4) Social prestige has a significant positive impact on the adoption of contour tillage technology by farmers in Gansu Province, with the standardized path coefficients of each observation variable being 0.858, 0.789, and 0.723, respectively, and with all of them passing the significance test. In addition, there is little difference in the degree of impact of each observation variable of social prestige on technology adoption by farmers in Gansu. However, social prestige has not significant impact in Shanxi and Shaanxi. One possible reason is that farmers with high social status tend to be elderly people who are highly respected in their villages. In the actual investigation, we found that most of these farmers are elderly people over the age of 60 years in Gansu, who have lived through the period of most serious soil erosion and can more easily recognize the effect of contour tillage technology, although they generally believe that it lacks small machinery. However, they are unwilling to abandon sloping and terraced land, and are willing to adopt contour tillage technology all the time. Simultaneously the speech and behavior of farmers with high social prestige will affect other farmers role model when the effect of technology adoption is better, helping to increase the rate of technology adoption.
(5) Social norms and technology cognition had significant positive effects on t adoption of contour tillage technology by farmers in the three provinces, with the standardized path coefficients of the observation variables in Shanxi being 0.934, 0.890, and 0.576; those in Shaanxi being 0.851, 0.878, and 0.894; and in Gansu being 0.639, 0.246, and 0.058, respectively, all passing the significance test. The farmers' behaviors are impacted by the attitudes and behaviors of people who surround them, especially relatives, friends, and villagers. When farmers see that the surrounding people have adopted the contour tillage technology, they feel pressured to get group affirmation with respect to the technology, since if they adopt this technology, they can obtain more resources and technology support.

## 5 Conclusions and implications for decision makers

### 5.1 Conclusions

From the perspective of regional differences, we explored the impact of social capital and technology cognition on the adoption of soil and water conservation tillage technology by farmers in three provinces of the Loess Plateau in China, based on a sample of 1237 farmers, using a multi-group structural equation model and taking contour tillage technology as an example. The main conclusions are as follows：

(1) From the perspective of the impact of social capital on the adoption of contour tillage technology by farmers, social network has a significant impact on the adoption of contour tillage technology by farmers in Shanxi, and Shaanxi Provinces social trust has a significant impact on the adoption of contour tillage technology by farmers in Shanxi, Shaanxi Provinces, and social participation has significant impact on the adoption of contour tillage technology by farmers in Shaanxi Province. Social prestige has a significant impact on the adoption of contour tillage technology in Gansu Province, and social norms have a significant impact on the adoption of contour tillage technology in the three provinces. There are significant regional differences in the impact of social capital on the adoption of contour tillage technology by farmers in the three provinces.
(2) Technology cognition has significant positive impact on the adoption of contour tillage technology by farmers in the three provinces, but the impact coefficient has significant regional differences. In the adoption of contour tillage technology by farmers in Shanxi Province technology cognition plays an intermediary role in the impact of social network social trust, and social norms on technology adoption. In the adoption of contour tillage technology by farmers in Shaanxi Province, technology cognition plays an intermediary role in the impact of social network, social trust and social norms on technology adoption. In the adoption of contour high tillage technology by farmers in Gansu Province, technology cognition plays mediating role in the impact of social norms on technology adoption. Meanwhile, there are significant regional differences among the three provinces in the intermediary role of technology cognition.
(3) Among the observed variables of social network, the frequency of contact between farmers and villagers has the greatest impact on the adoption of contour tillage technology in Shanxi and Shaanxi provinces, which indicates that farmers' technology adoption behavior in the two provinces is most affected by villagers. The effect of imitation is obvious. Among the observed variables of social trust, the degree of farmers' trust in the villagers of Shanxi and Shaanxi. Among the observation variables of social participation, participation in collective activities in villages has the greatest impact on the adoption of contour tillage technology by farmers in Shaanxi Province, and the observation variables of social prestige have little impact on the adoption of technology by farmers in Gansu Province. In the social norms, the attitude and behavior of relatives, friends and villagers toward contour tillage technology have a greater impact on farmers.

### 5.2 Implications for decision makers

Based on the study conclusions, we put offer the following suggestions. First, under the current situation of the acquaintance society in China, farmers obtain information and resources from the social capital. Thus, social capital is a key factor affecting the farmers' adoption of soil and water conservation tillage technology. Therefore, to encourage farmers to adopt soil and water conservation technology, it is necessary to build a multi-dimensional social networks to enhance mutual trust among the networks members. Second, the government should promote soil and water conservation policies and technology, and raise the farmers' awareness, so that farmers can fully realize the economic and ecological benefits of the contour tillage technology. Moreover, through various forms of subsidies, the government should reduce the cost of technology adoption and improve the degree of farmers’ adoption. Third, farmers with high social prestige can play an exemplary and leading role in technology promotion. Simultaneously, farmers should be encouraged to participate in the village collective affairs and close community relations.

## Funding

The authors gratefully acknowledge the financial supports by the Shanxi Province science and technology strategy research project (202,204,031,401,091). National Social Science Foundation of China (21BMZ082)

## Data availability statement

Data will be made available on request.

## CRediT authorship contribution statement

**Liu Li:** Formal analysis, Data curation, Conceptualization. **Shangguan Dingyi:** Supervision, Resources, Methodology, Investigation, Data curation. **Sun Fengluan:** Investigation, Formal analysis. **Tai Xiujun:** Supervision, Resources, Investigation, Data curation. **Hafeez Noor:** Writing – review & editing, Writing – original draft, Funding acquisition.

## Declaration of competing interest

The authors declare that they have no known competing financial interests or personal relationships that could have appeared to influence the work reported in this paper.
